# Supplementary material for: Ultralow-voltage operation of light-emitting diodes
Source: Nat Commun. 2022 Jul 4;13:3845. doi: 10.1038/s41467-022-31478-y (PMC9253117; doi:10.1038/s41467-022-31478-y)
Supplement: Supplementary file 1 — Supplementary Information [file 41467_2022_31478_MOESM1_ESM.pdf]

Supplementary Information for:

Ultralow-voltage operation of light-emitting diodes

Yaxiao Lian[1#], Dongchen Lan[2,3#], Shiyu Xing[1#], Bingbing Guo[1], Zhixiang Ren[1], Runchen Lai[1], Chen Zou[1], Baodan Zhao[1,4], Richard H. Friend[4], Dawei Di[1,4*]

1. State Key Laboratory of Modern Optical Instrumentation, College of Optical Science and Engineering; International Research Center for Advanced Photonics, Zhejiang University, Hangzhou, 310027, China
2. College of Electrical Engineering, Zhejiang University, Hangzhou, 310027, China
3. Australian Centre for Advanced Photovoltaics, University of New South Wales, Sydney, 2052, Australia
4. Cavendish Laboratory, University of Cambridge, JJ Thomson Avenue, Cambridge, CB3 0HE, United Kingdom

[#] These authors contributed equally to this work.

[*]Corresponding author. E-mail: daweidi@zju.edu.cn

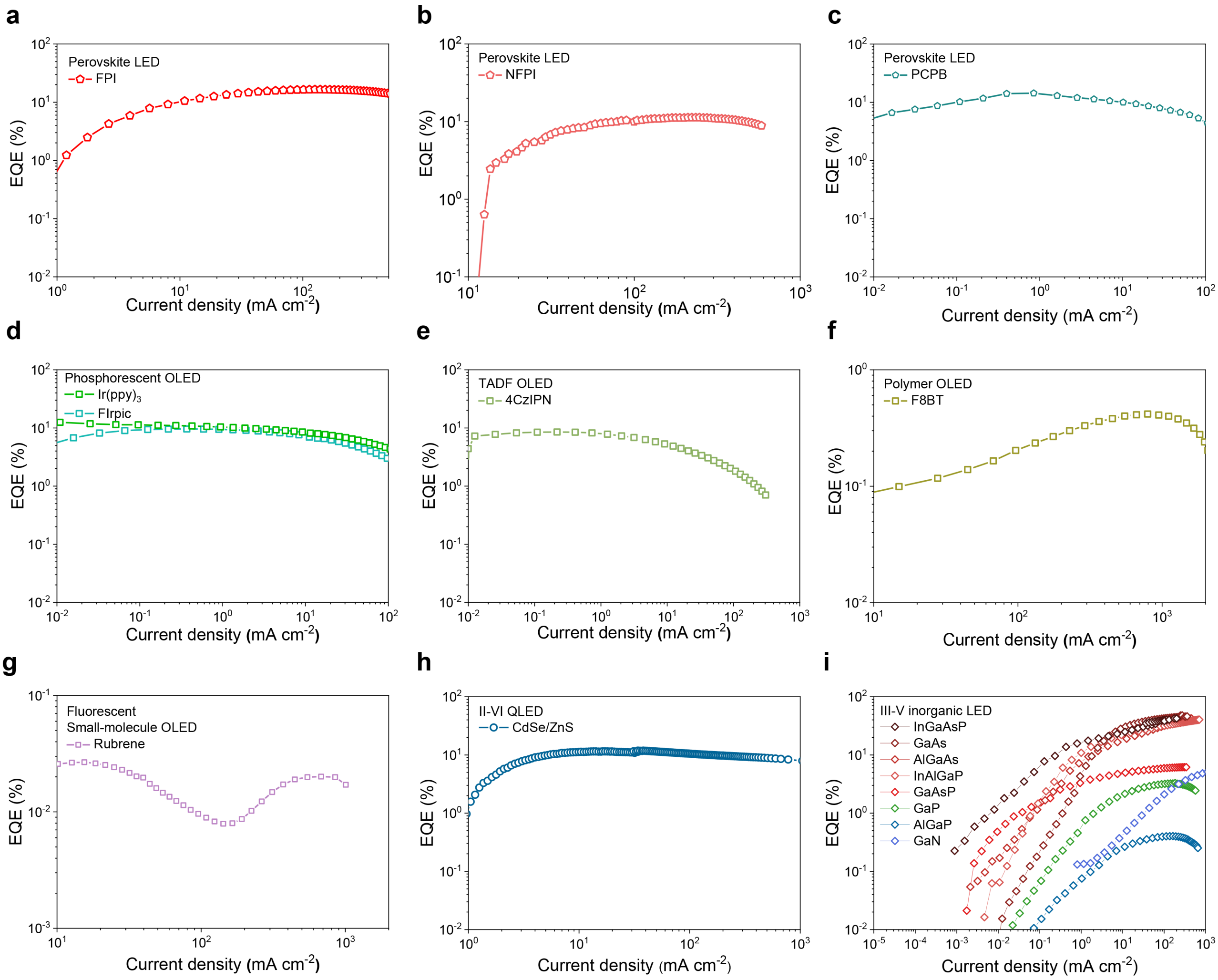

Supplementary Figure 1 | EQE versus current density curves for different classes of LEDs. a, NIR-emitting FPI perovskite LED. **b,** NIR-emitting NFPI perovskite LED. **c,** Green-emitting PCPB perovskite LED. **d,** Phosphorescent OLEDs based on Ir(ppy)$_3$ and FIrpic. **e,** TADF OLED based on 4CzIPN. **f,** Polymer OLED based on F8BT. **g,** Fluorescent small-molecule OLED based on rubrene, prepared using a host-free configuration. The light-emitting performance of the host-free rubrene layer is limited by the PEDOT:PSS and C60 layers which are exciton quenchers, leading to the significantly reduced EQEs. **h,** II-VI QLED based on CdSe/ZnS QDs. **i,** Commercial III-V inorganic LEDs based on InGaAsP, GaAs, AlGaAs, InAlGaP, GaAsP, GaP, AlGaP and GaN.

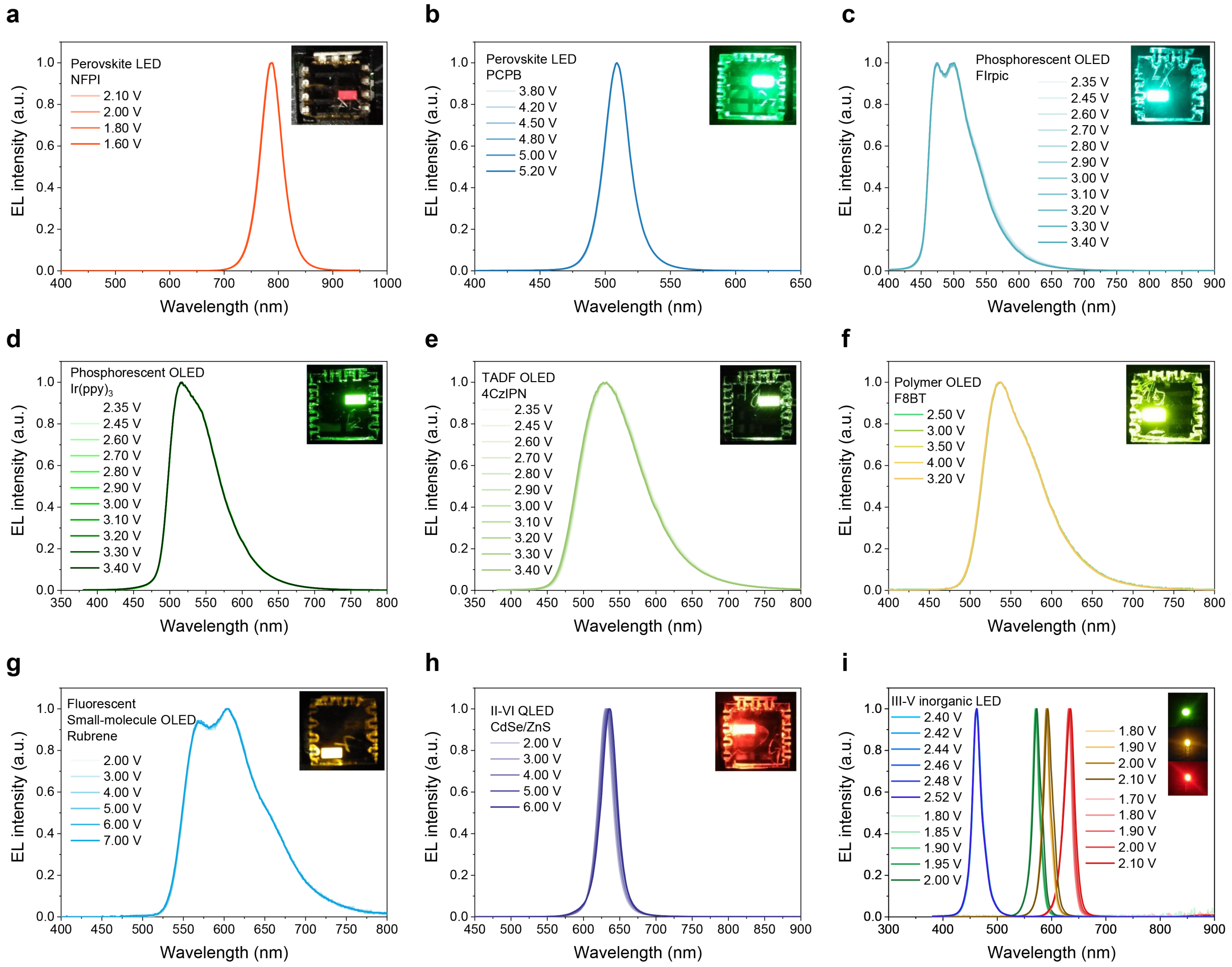

Supplementary Figure 2 | Normalized EL spectra of different classes of LEDs under different operating voltages. a, Perovskite LED based on NFPI. **b,** Perovskite LED based on PCPB. **c,** Phosphorescent OLED based on FIrpic. **d,** Phosphorescent OLED based on $Ir(ppy)_3$. **e,** TADF OLED based on 4CzIPN. **f,** Polymer OLED based on F8BT. **g,** Fluorescent small-molecule OLED based on rubrene. **h,** II-VI QLED based on CdSe/ZnS QDs. **i,** Commercial III-V inorganic LEDs based on GaAsP, GaP, AlGaP and GaN. Insets are photos of working LEDs.

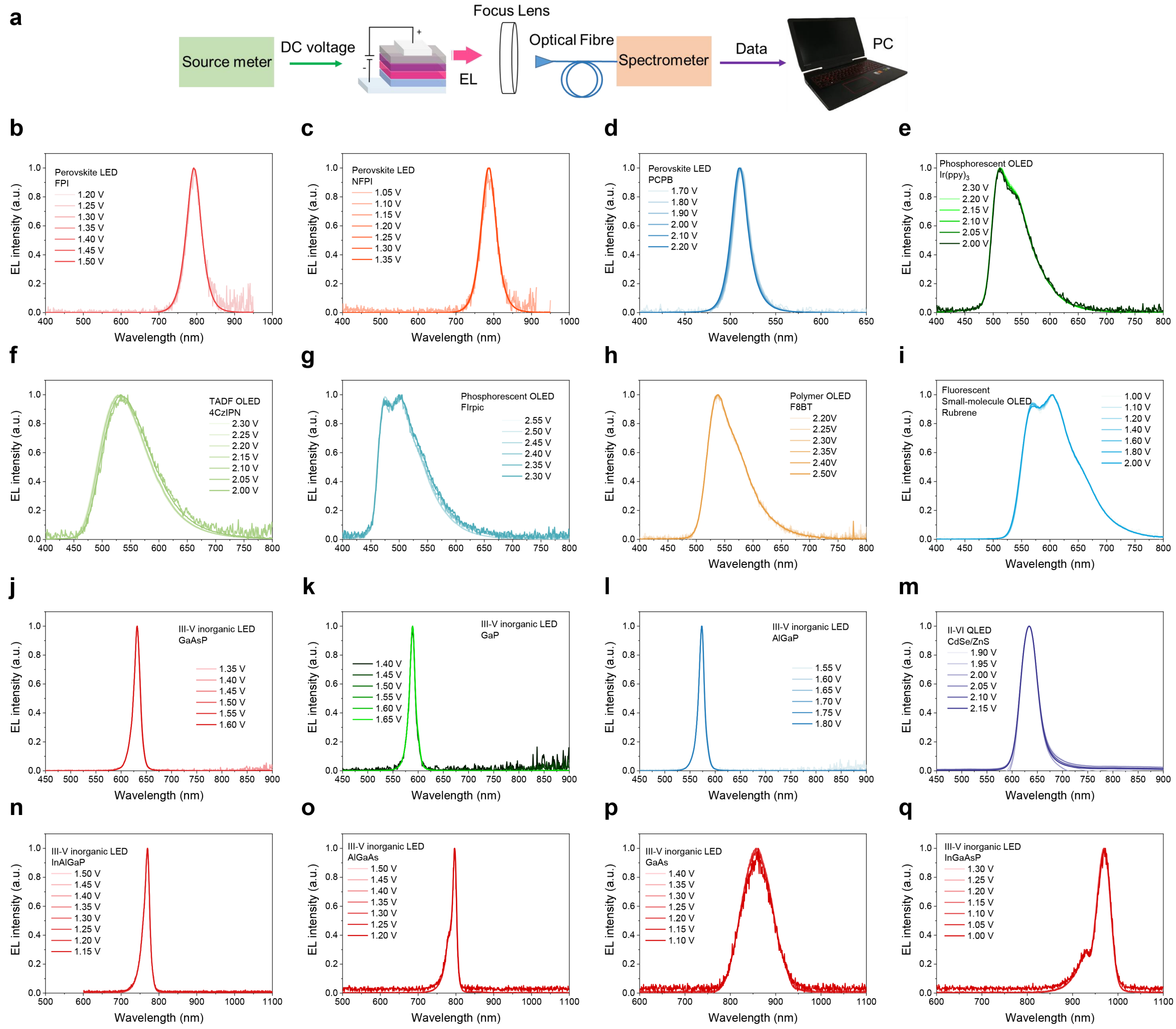

Supplementary Figure 3 | Normalized EL spectra of different classes of LEDs under sub-bandgap voltages. a, Schematic of experimental setups for measuring EL spectra operated at sub-bandgap voltages. **b,** Perovskite LED based on FPI. **c,** Perovskite LED based on NFPI. **d,** Perovskite LED based on PCPB. **e,** Phosphorescent OLED based on Ir(ppy)$_3$. **f,** TADF OLED based on 4CzIPN. **g,** Phosphorescent OLED based on FIrpic. **h,** Polymer OLED based on F8BT. **i,** Fluorescent small-molecule OLED based on rubrene. **j–l,** Commercial III-V inorganic LEDs based on GaAsP, GaP, and AlGaP. **m,** II-VI QLED based on CdSe/ZnS QDs. **n–q**, Commercial III-V inorganic LEDs based on InAlGaP, AlGaAs, GaAs, and InGaAsP.

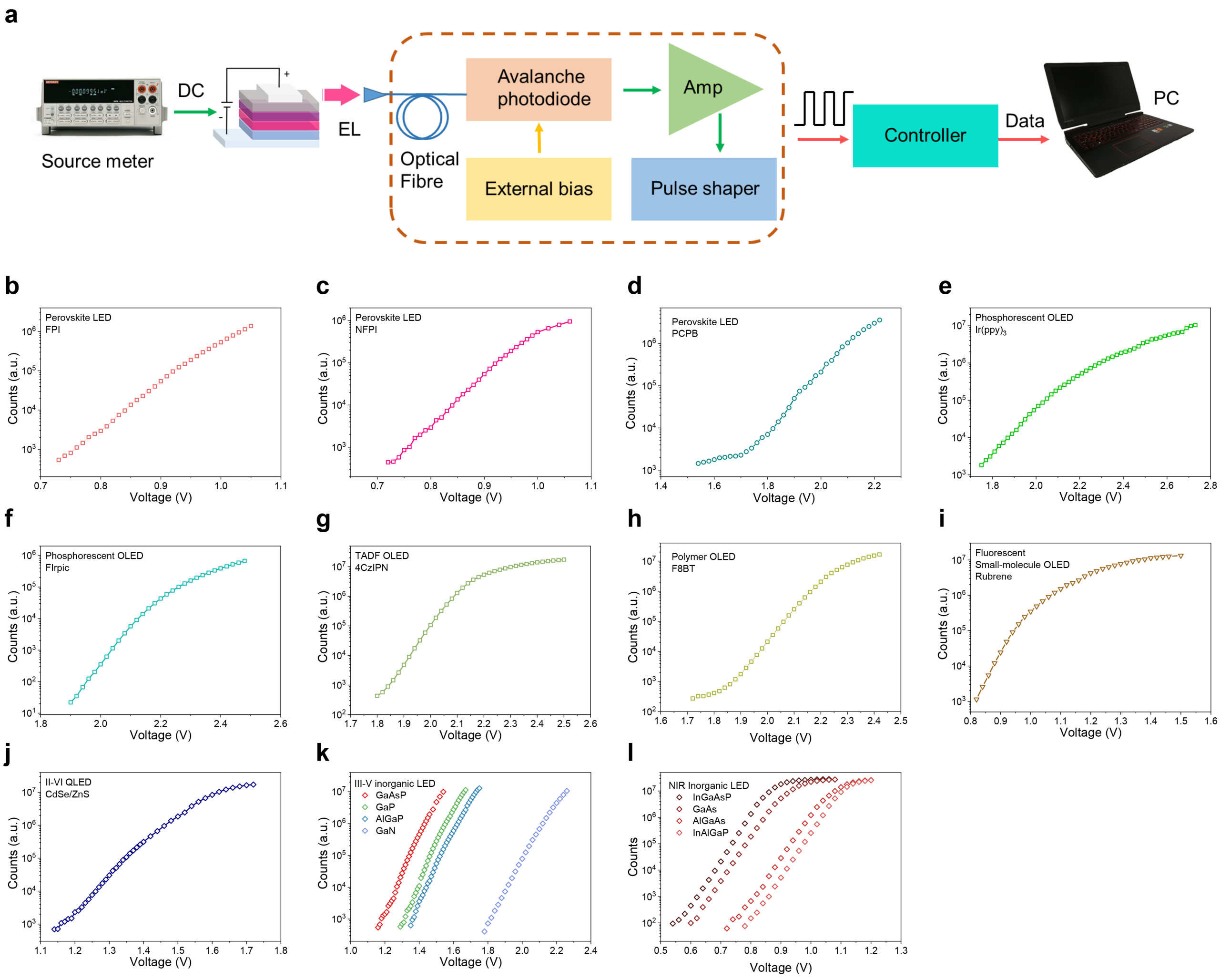

Supplementary Figure 4 | High-sensitivity photon detection experiments. a, Schematic of the high-sensitivity photon detection system. The original APD counts-voltage characteristics of: **b**, FPI perovskite LED; **c**, NFPI perovskite LED; **d**, PCPB perovskite LED; **e**, Phosphorescent OLED based on Ir(ppy)$_3$; **f**, Phosphorescent OLED based on FIrpic; **g**, TADF OLED based on 4CzIPN; **h**, Polymer OLED based on F8BT; **i**, Fluorescent small-molecule OLED based on rubrene; **j**, II-VI QLED based on CdSe/ZnS QDs; **k**, Commercial III-V inorganic LEDs based on GaAsP, GaP, AlGaP, and GaN; **l**, Commercial III-V inorganic LEDs based on InAlGaP, AlGaAs, GaAs, and InGaAsP.

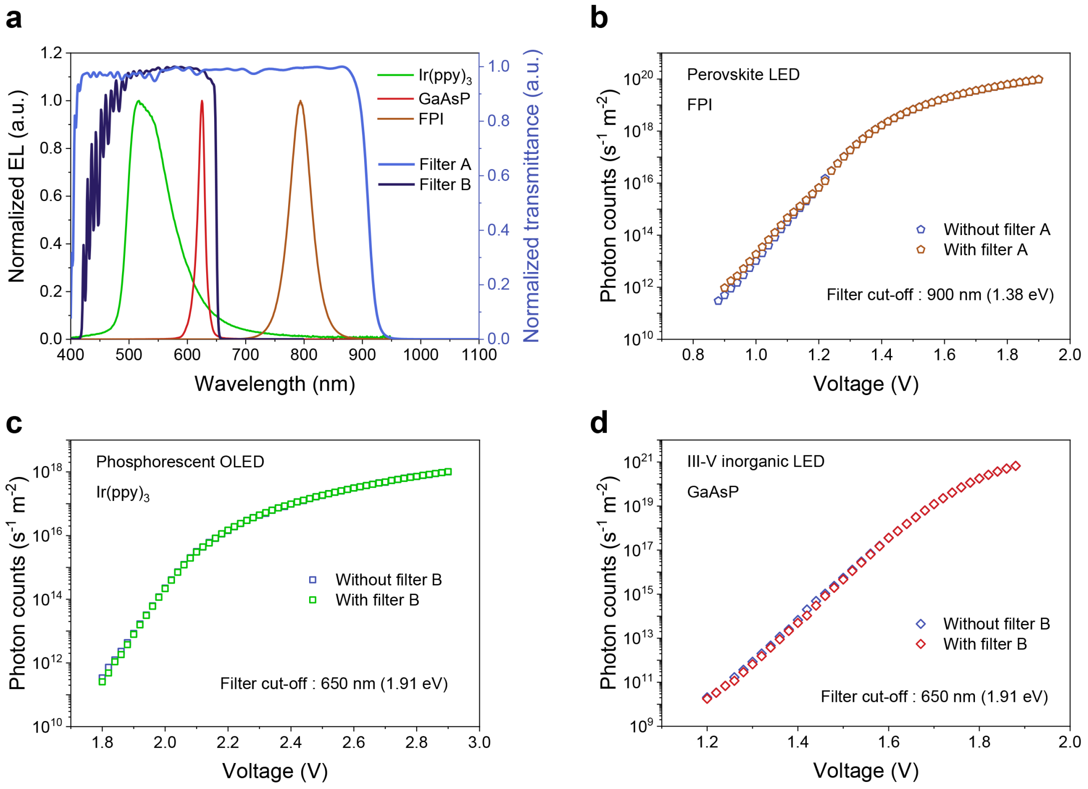

Supplementary Figure 5 | EL measurements with and without bandpass filters. a, EL spectra of LEDs and the transmission spectra of the bandpass filters. **b,** EL intensity-voltage characteristics of FPI perovskite LEDs. **c,** EL intensity-voltage characteristics of $Ir(ppy)_3$ OLEDs. **d,** EL intensity-voltage characteristics of GaAsP LEDs.

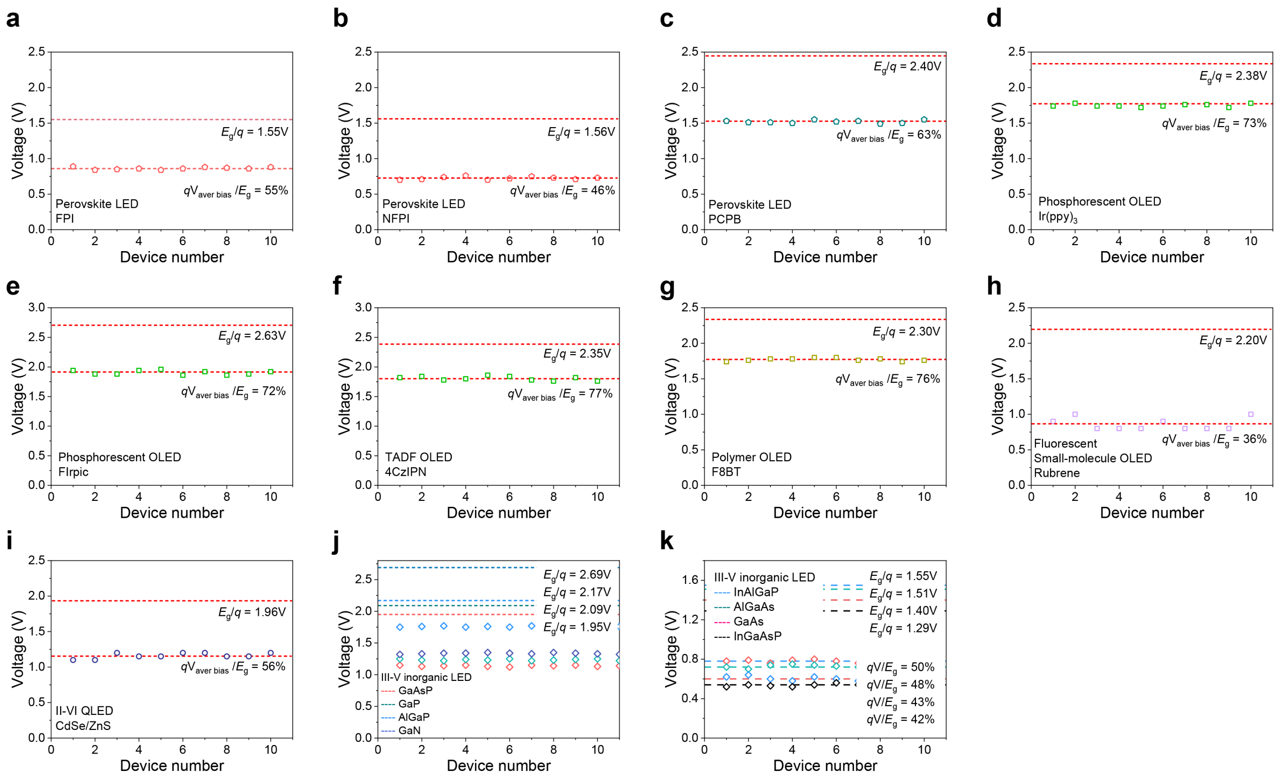

Supplementary Figure 6 | Statistics of the measured minimum operating voltages for different classes of LEDs. a, Perovskite LED based on FPI. **b,** Perovskite LED based on NFPI. **c,** Perovskite LED based on PCPB. **d,** Phosphorescent OLED based on Ir(ppy)3. **e,** Phosphorescent OLED based on Firpic. **f,** TADF OLED based on 4CzIPN. **g,** Polymer OLED based on F8BT. **h,** Fluorescent small-molecule OLED based on rubrene. **i,** II-VI QLED based on CdSe/ZnS QDs. **j,** Commercial III-V inorganic LEDs based on GaN, GaAsP, GaP, and AlGaP. **k,** Commercial III-V inorganic LEDs based on InAlGaP, AlGaAs, GaAs, and InGaAsP.

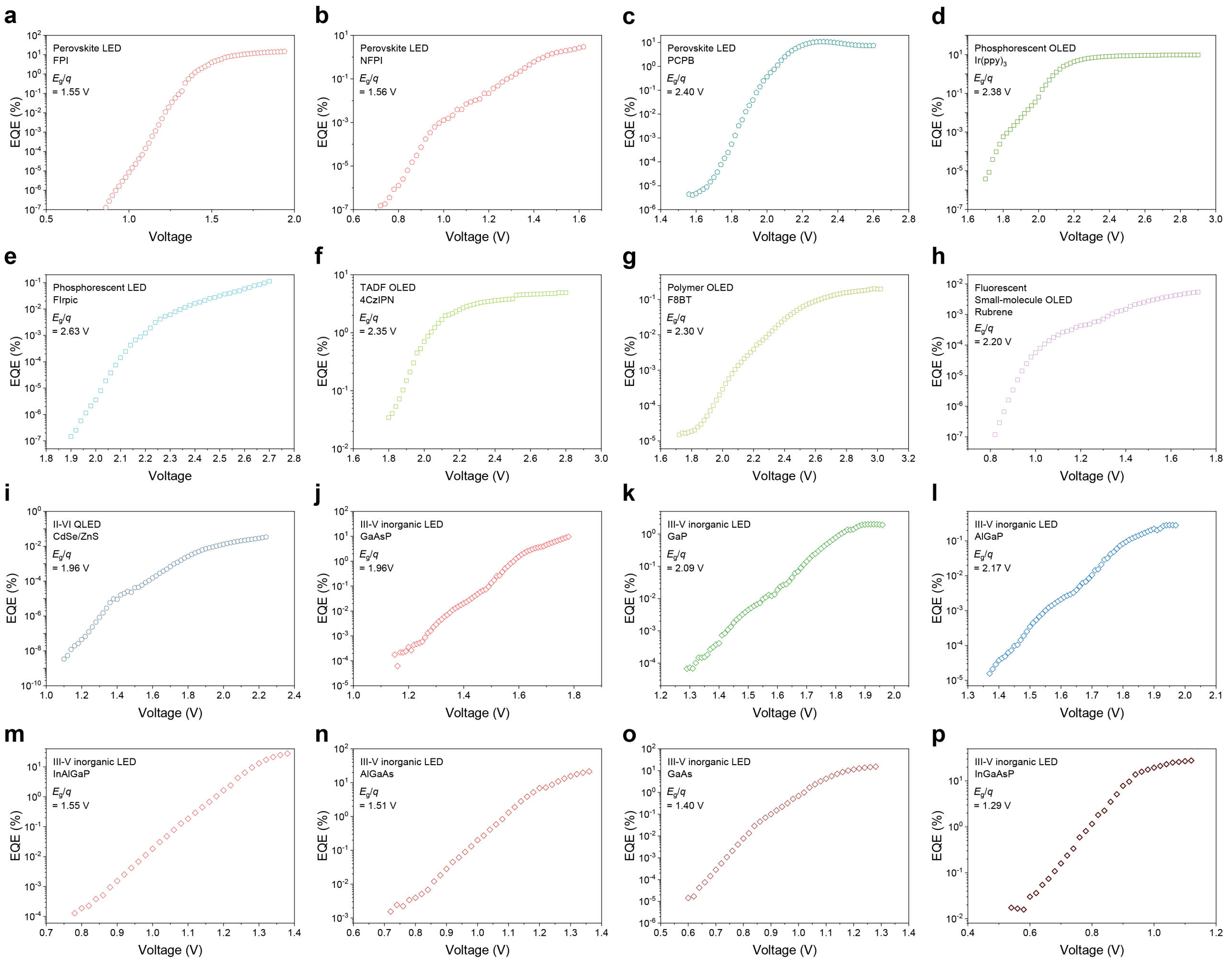

Supplementary Figure 7 | The EQE-voltage curves for different classes of LEDs measured using a high-sensitivity photon detection system. a, Perovskite LED based on FPI. **b,** Perovskite LED based on NFPI. **c,** Perovskite LED based on PCPB. **d,** Phosphorescent OLED based on Ir(ppy)$_3$. **e,** Phosphorescent OLED based on fIrpic. **f,** TADF OLED based on 4CzIPN. **g,** Polymer OLED based on F8BT. **h,** Fluorescent small-molecule OLED based on rubrene. **i,** II-VI QLED based on CdSe/ZnS QDs. **j-p,** Commercial III-V inorganic LEDs based on GaAsP, GaP, AlGaP, InAlGaP, AlGaAs, GaAs, and InGaAsP.

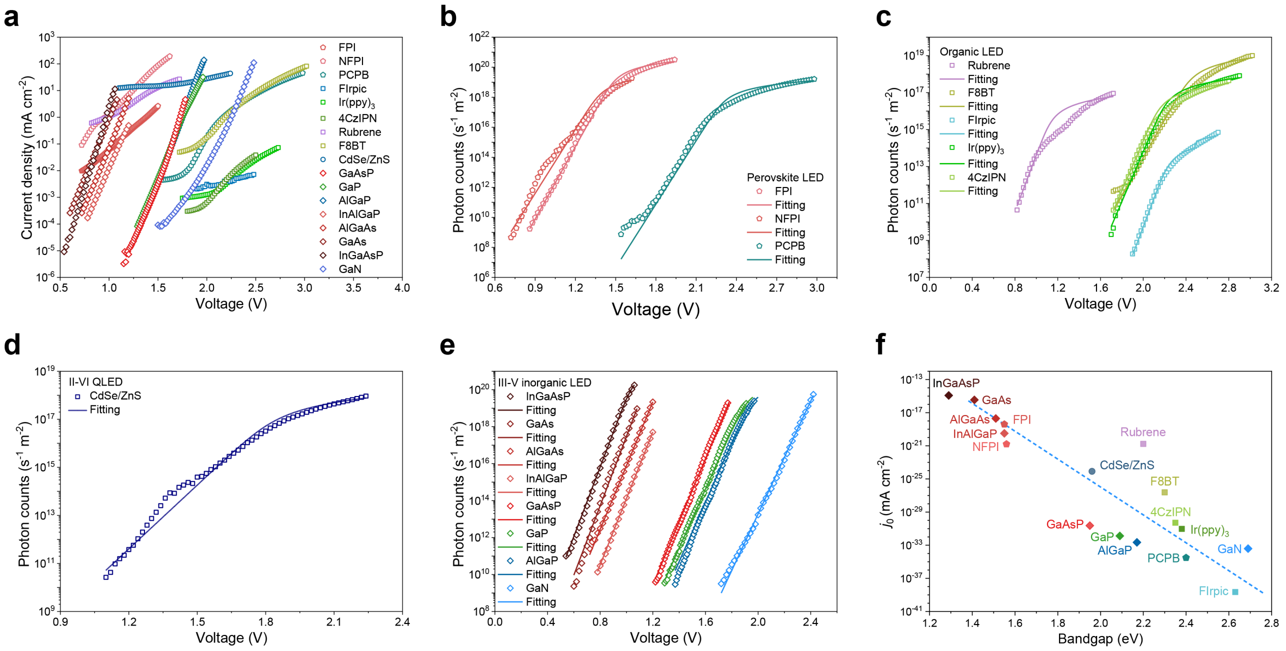

Supplementary Figure 8 | Current-voltage and EL intensity-voltage characteristics measured with high-sensitivity photon detection system. a, Current density-voltage curves of different classes of LEDs. **b,** EL intensity-voltage data and curve fitting of perovskite LEDs based on FPI, NFPI and PCPB. **c,** EL-voltage data and curve fitting of OLEDs based on Ir(ppy)$_3$, fIrpic, 4CzIPN, F8BT and rubrene. **d,** EL intensity-voltage data and curve fitting of II-VI QLED based on CdSe/ZnS. **e,** EL-voltage data and curve fitting of commercial III-V inorganic LEDs based on GaN, GaAsP, GaP, AlGaP, InAlGaP, AlGaAs, GaAs, InGaAsP. **f,** j_0 versus E_g for different classes of LEDs.

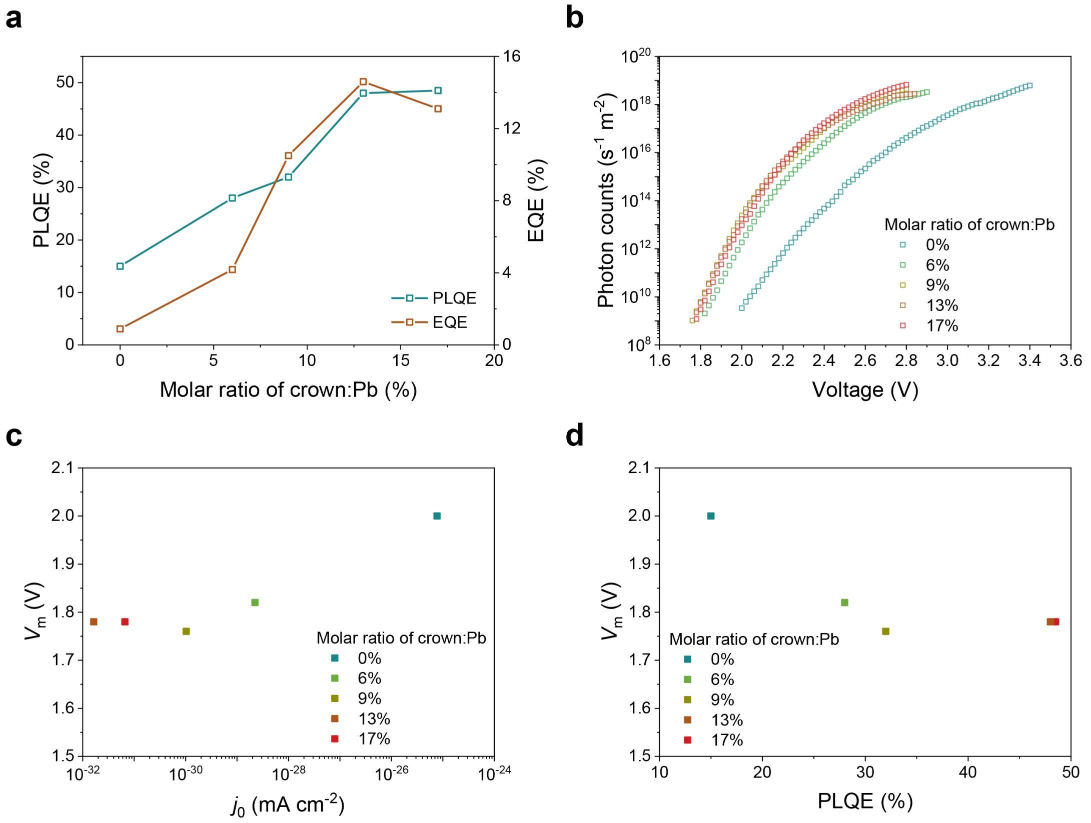

Supplementary Figure 9 | Characteristics of PCPB perovskite LEDs with different molar fractions of molecular additives. The molecular additive used was 1,4,7,10,13,16-hexaoxacyclooctadecane (crown). The molar ratio of the crown additive versus Pb in the precursor solution was tuned from 0%-17%. **a,** PLQEs of perovskite films, and EQEs of perovskite LEDs based on the same compositions as for the PLQE experiments. **b,** EL intensity-voltage characteristics. **c,** V_m versus j_0. **d,** V_m versus peak EQE.

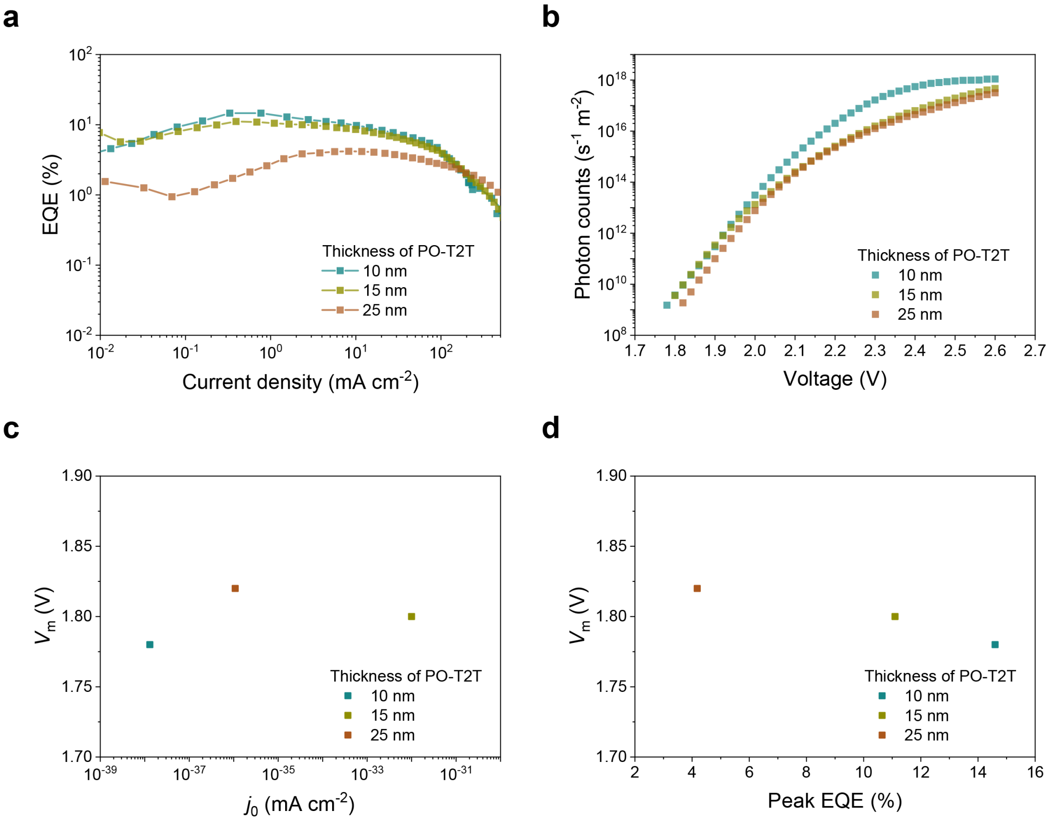

Supplementary Figure 10 | Characteristics of PCPB perovskite LEDs with electron-transpot layer (ETL) thickness variation. The ETL used was 2,4,6-tris[3-(diphenylphosphinyl)phenyl]-1,3,5-triazineis (PO-T2T). **a,** EQE-current density curves. **b,** EL intensity-voltage characteristics. **c,** V_m versus j_0. **d,** V_m versus peak EQE.

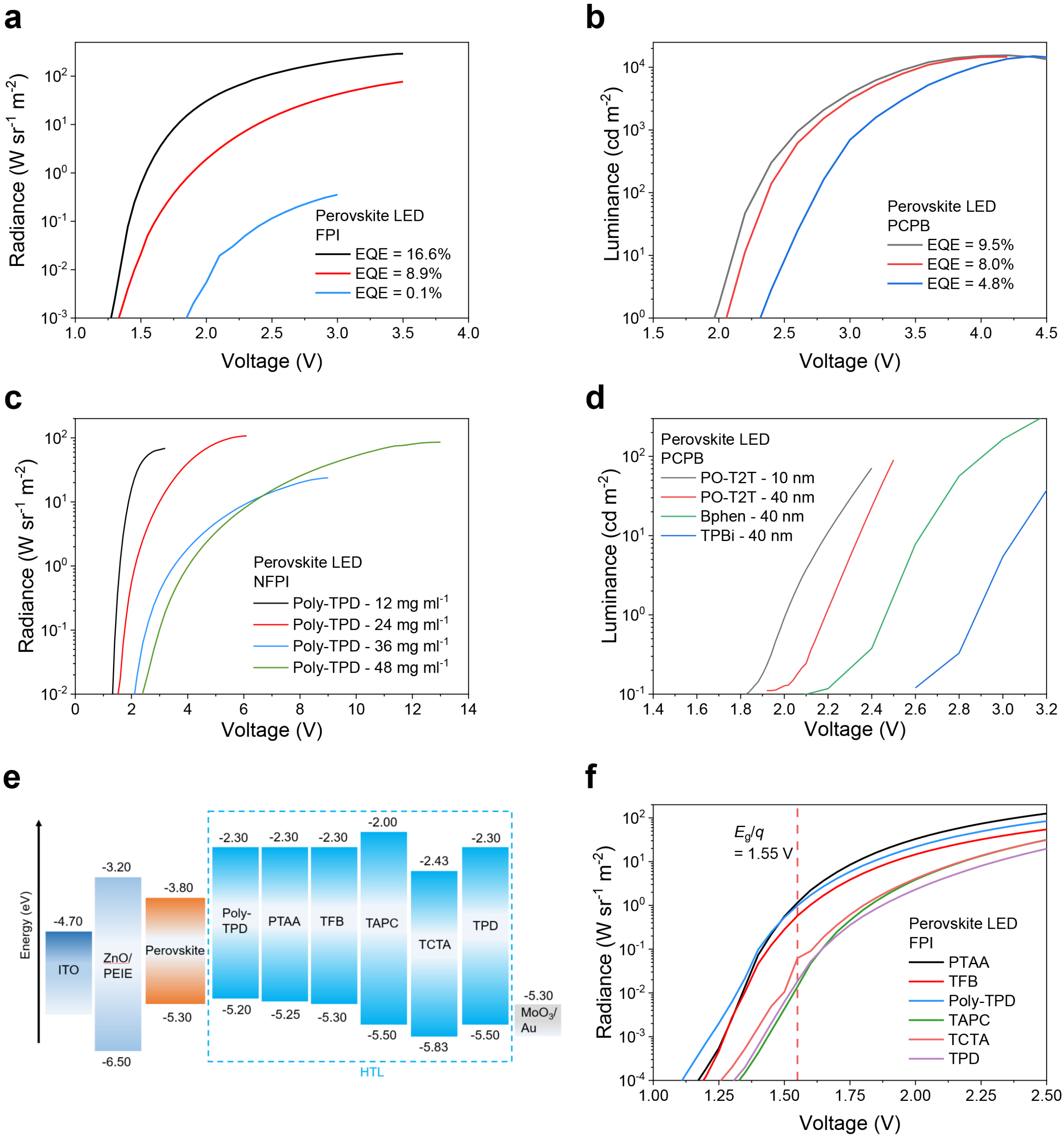

Supplementary Figure 11 | EL performance of PeLEDs with different charge-transport layers. a, Radiance-voltage characteristics of FPI perovskite LEDs with different EQEs. **b,** Luminance-voltage characteristics of PCPB perovskite LEDs with different EQEs. **c,** Radiance-voltage characteristics of NFPI perovskite LEDs with HTLs prepared from poly-TPD solutions with different concentrations. **d,** Luminance-voltage characteristics of PCPB perovskite LEDs with different ETLs. **e,** Energy level diagrams for FPI perovskite LEDs with various HTLs. **f,** Radiance-voltage characteristics of FPI perovskite LEDs with different HTLs.

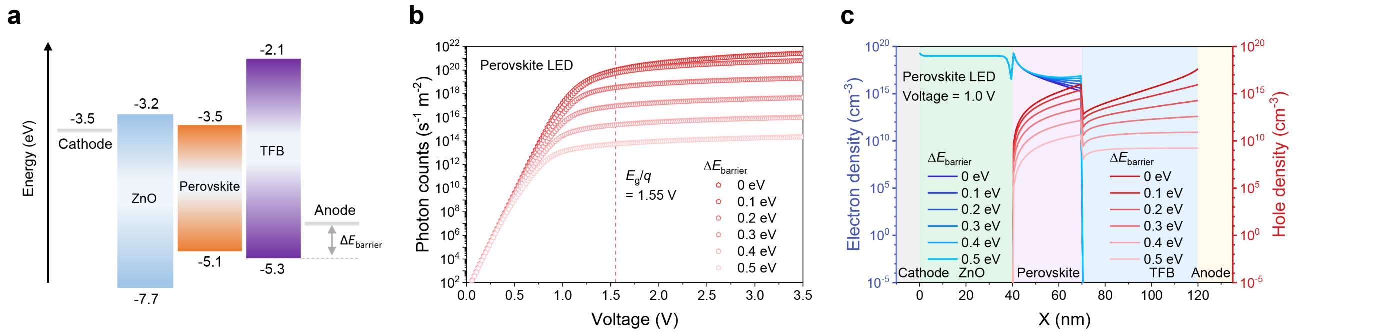

Supplementary Figure 12 | Simulated EL intensity-voltage characteristics and carrier density distributions for lead iodide PeLEDs with variable hole-injection barriers. a, Energy level diagram of a lead iodide perovskite LED with a variable anode/HTL barrier ($\Delta E_{\mathrm{barrier}}$). $\Delta E_{\mathrm{barrier}} = |E_{\mathrm{HOMO,TFB}}| - |E_{f,\mathrm{anode}}|$, where $E_{f,\mathrm{anode}}$ is the Fermi level of the anode, and $E_{\mathrm{HOMO,TFB}}$ is the highest occupied molecular orbital (HOMO) level of the TFB HTL. **b,** Effect of the anode/HTL barrier height on the EL intensity-voltage characteristics. **c,** Effect of the anode/HTL barrier height on carrier density distribution in a lead iodide perovskite LED (E_g = 1.55 eV) under a sub-bandgap driving voltage of 1 V. The shaded regions in gray, light green, pink, light blue and yellow correspond to cathode, ZnO, perovskite, TFB and anode, respectively.

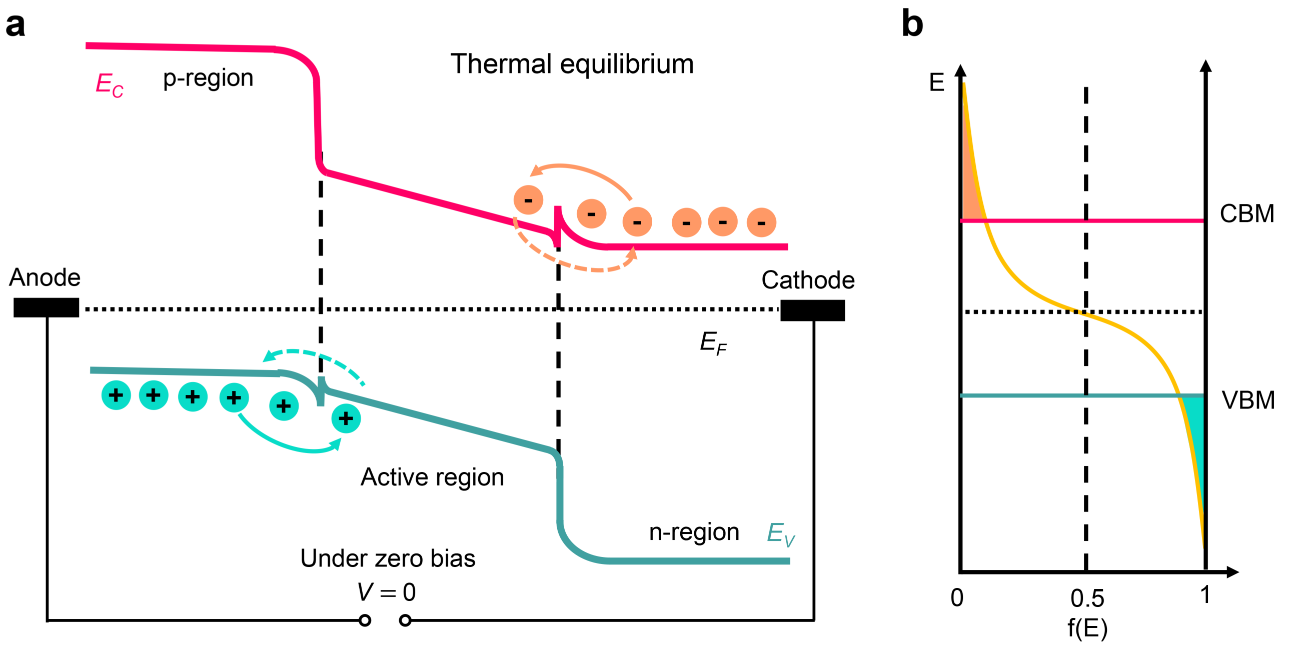

Supplementary Figure 13 | Illustration of a generic heterojunction diode under thermal equilibrium condition (which does not generate EL). a, Energy band diagram of a heterojunction LED under zero bias where the drift current represented by the dashed arrow equals the diffusion current represented by the solid arrow). **b,** Schematic diagram of free electrons (orange solid fill) and holes (green solid fill) distributions near the band edges in the active region, where *f*(E) is the Fermi-Dirac distribution function.

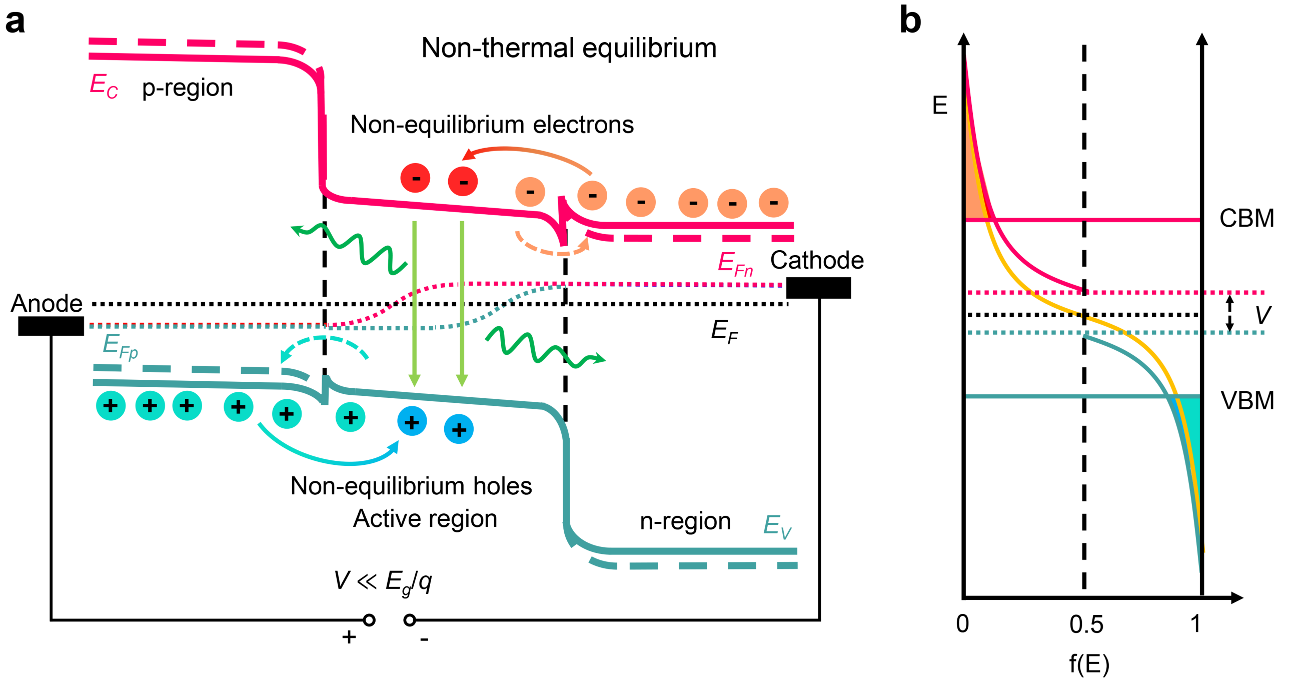

Supplementary Figure 14 | Illustration of EL photon emission from a generic heterojunction diode under a very small forward bias. a, Energy band diagram of a heterojunction LED under a non-zero forward bias. **b,** Schematic diagram of free electrons (orange solid fill), non-thermal-equilibrium electrons (red solid fill), holes (green solid fill), non-thermal-equilibrium holes (blue solid fill) distributions near the band edges in the active region, where *f*(E) is the Fermi-Dirac distribution function.

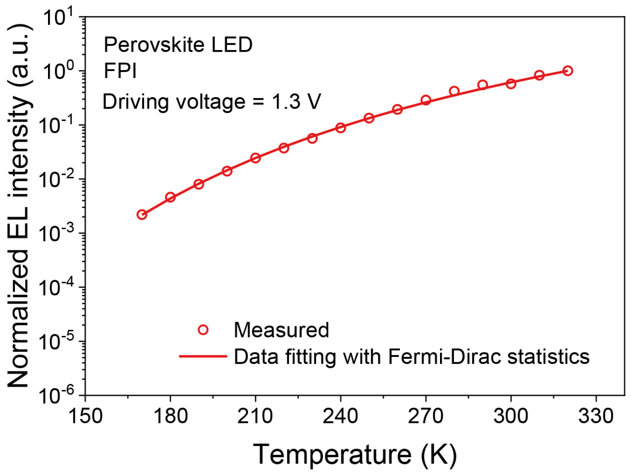

Supplementary Figure 15 | EL intensity-temperature characteristics of a FPI perovskite LED. The EL intensities at different temperatures are normalized to unity at the highest temperature. The measured EL intensity (I_{EL})-temperature data can be fitted satisfactorily according to $I_{EL} \propto \int_{E_g}^{\infty} f(E)\, N_j dE$, where E_g is the bandgap, and $f(E) = \frac{1}{1+e^{\frac{[E-(E_{fe}-E_{fh})]}{kT}}}$, which gives the distribution of carriers governed by the Fermi-Dirac function. E_{fe} and E_{fh} are the quasi-Fermi levels of electrons and holes; the quasi-Fermi level splitting E_{fe}-E_{fh} is determined by the driving voltage. k is the Boltzmann constant; T is the temperature. $N_j(E) = \frac{m_r^*}{\pi^2 \hbar^3}\sqrt{E - E_g}$ is the joint density of states; m_r^* is the reduced effective mass defined by $\frac{1}{m_r^*} = \frac{1}{m_e^*} + \frac{1}{m_h^*}$, where m_e^* and m_h^* are the effective masses of electrons and holes, respectively[1].

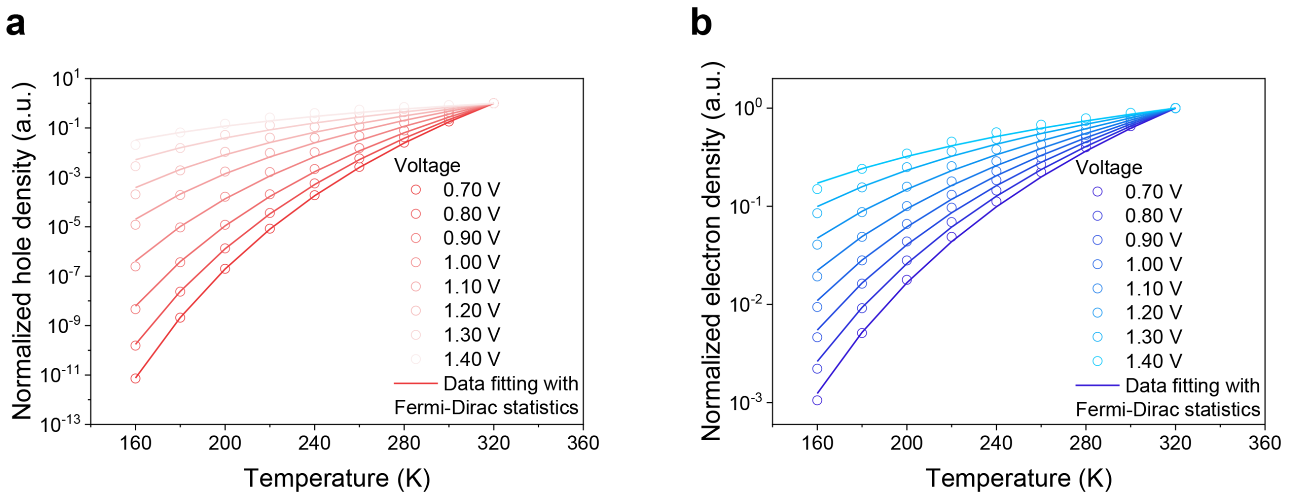

Supplementary Figure 16 | Simulated carrier density-temperature characteristics of a lead iodide perovskite LED. The carrier densities at different temperatures are normalized to unity at the highest temperature. In these simulations, the carrier densities at the centre of the emissive layer are taken as the representative data p. **a,** Hole density-temperature characteristics for different driving voltages. **b,** Electron density-temperature characteristics for different driving voltages. The data can be fitted satisfactorily using the Fermi-Dirac statistics. The density of electrons, $n_e \propto \int_{E_g}^{\infty} \frac{1}{1+e^{\frac{(E-E_{fe})}{kT}}} \sqrt{E-E_g} dE$. The density of holes, $n_h \propto \int_{E_g}^{\infty} \frac{1}{1+e^{\frac{(E-E_{fh})}{kT}}} \sqrt{E-E_g} dE$. Here, E_{fe} and E_{fh} are the quasi-Fermi levels of electrons and holes; The quasi-Fermi level splitting E_{fe}-E_{fh} is determined by the driving voltage; E_g is the bandgap; k is the Boltzmann constant; T is the temperature.

Supplementary Table 1 | Measured minimum operating voltages of different LEDs from the literature.

Device type	Emissive material	Peak wavelength (nm)	Bandgap (eV)	Minimum voltage (V)	qVm/Eg	Ref.
Perovskite LED	$(NMA)_2(FA)_{n-1}Pb_nI_{3n+1}$	795	1.56	1.25	80%	13
Perovskite LED	$(NMA)_2FAPb_2I_7$	786	1.58	1.5	95%	40
Perovskite LED	$CsPbBr_3$	525	2.36	1.9	81%	42
Polymer OLED	F8BT	538	2.3	2.3	100%	45
Fluorescent small-molecule OLED	Rubrene	563	2.2	1.0	46%	30
II-VI QLED	CdSe/CdZnS	600	2.1	1.7	81%	10
II-VI QLED	CdSe/CdS	640	1.94	1.7	88%	11
II-VI QLED	CdSe-based	620	2.0	1.6	80%	26
III-V inorganic LED	$In_{0.2}Ga_{0.8}As$	953	1.3	0.9	70%	35

Supplementary Table 2 | Dark saturation current densities (j_0) and the corresponding $j_0 e^{\frac{E_g}{kT}}$ of different classes of LEDs.

Device type	Emissive material	Ideality factor, n	j_0 (mA cm^{-2})	$j_0\exp(E_g/kT)$ (mA cm^{-2})
Perovskite LED	$FAPbI_3$ (FPI) perovskite	1.05	4.0×10^{-19}	3.9×10^{7}
Perovskite LED	NFPI perovskite	1.30	1.6×10^{-21}	2.3×10^{5}
Perovskite LED	PCPB perovskite	1.22	3.1×10^{-35}	7.5×10^{1}
Phosphorescent OLED	$Ir(ppy)_3$	1.25	9.3×10^{-32}	7.5×10^{8}
Phosphorescent OLED	FIrpic	1.1	2.2×10^{-39}	2.8×10^{5}
TADF OLED	4CzIPN	1.25	5.1×10^{-31}	1.3×10^{9}
Polymer OLED	F8BT	1.65	2.4×10^{-27}	8.9×10^{11}
Fluorescent small-molecule OLED	Rubrene	1.10	1.7×10^{-21}	1.3×10^{16}
II-VI QLED	CdSe/ZnS	1.32	8.2×10^{-25}	6.0×10^{8}
III-V inorganic LED	GaN	1.09	3.9×10^{-34}	5.0×10^{11}
III-V inorganic LED	GaAsP	1.00	2.3×10^{-31}	1.2×10^{2}
III-V inorganic LED	GaP	1.03	1.3×10^{-32}	1.5×10^{3}
III-V inorganic LED	AlGaP	1.00	2.2×10^{-33}	5.4×10^{3}
III-V inorganic LED	InAlGaP	1.00	3.2×10^{-20}	2.2×10^{5}
III-V inorganic LED	AlGaAs	1.00	2.0×10^{-18}	1.8×10^{7}
III-V inorganic LED	GaAs	1.00	3.5×10^{-16}	1.7×10^{6}
III-V inorganic LED	InGaAsP	1.00	1.2×10^{-15}	1.7×10^{5}

Supplementary Table 3 | The settings and parameters for the simulation of lead iodide perovskite LED using Setfos.

Device structure: glass/cathode/ZnO/perovskite/TFB/anode.

**Layer**	**Thickness (nm)**	**Energy level (eV)**	**Mobility ($cm^2\ V^{-1}\ s^{-1}$)**	**Dielectric constant (relative permittivity)**	**Other parameters**
Glass	5×10^6	-	-	-	Refractive index: 1.55
Cathode	100	Work function: 3.3	-	-	-
ZnO	40	VBM: -7.7<br>CBM: -3.2	Electron mobility: 10^{-4}	3.5	Mobility of anions and cations: 10^{-6} $cm^2/V \cdot s$<br>Doping density: 10^{25} m^{-3}<br>Trap density: 10^{21} m^{-3}<br>Trap energy depth: 0.4 eV
perovskite	30	VBM: -5.1<br>CBM: -3.5	Electron and hole mobility: 10^{-6}	3.5	Mobility of anions and cations: 10^{-6} $cm^2/V \cdot s$<br>Dipole orientation: 0.23<br>Generation efficiency: 1
TFB	50	HOMO: -5.3<br>LUMO: -2.1	Hole mobility: 10^{-4}	3.5	Mobility of anions and cations: 10^{-6} $cm^2/V \cdot s$
Anode	100	Work function: 5.3	-	-	-

Supplementary Table 4 | The settings and parameters for the simulation of Ir(ppy)$_3$ OLED using Setfos. Device structure: glass/anode/PEDOT:PSS/CBP:Ir(ppy)$_3$/TPBi/cathode.

Layer	Thickness (nm)	Energy level (eV)	Mobility (cm^2 V^{-1} s^{-1})	Dielectric constant	Other parameters
Glass	5×10^6	-	-	-	Refractive index: 1.55
Anode	100	Work function: 5.3	-	-	-
PEDOT:PSS	30	HOMO: -5.5<br>LUMO: -3.6	Hole mobility: 8×10^{-3}	3.5	Mobility of anions and cations: 10^{-6} $cm^2/V \cdot s$<br>Doping density: 10^{26} m^{-3}
CBP:Ir(ppy)$_3$	20	HOMO: -5.6<br>LUMO: -3.0	Electron and hole mobility:10^{-6}	3.5	Mobility of anions and cations: 10^{-6} $cm^2/V \cdot s$<br>Dipole orientation: 0.23<br>Generation efficiency: 1
TPBi	40	HOMO: -6.3<br>LUMO: -2.7	Electron mobility: 10^{-5}	3.5	Mobility of anions and cations: 10^{-6} $cm^2/V \cdot s$
Cathode	100	Work function: 2.7	-	-	-

Supplementary Table 5 | Energy consumption of a perovskite LED working as a photon source in the optical transmitter setup. The perovskite LED was driven by electrical pulses with a peak voltage of 1 V (qV/E_g = 65%).

**Frequency (Hz)**	**Current (mA cm^{-2})**	**FWHM of EL pulses (ns)**	**Energy (pJ/bit)**
100	8	15.3	147
1,000	7.5	15.4	138
10,000	7.6	15.3	139
100,000	7.4	15.3	136
1,000,000	7.8	15.2	142

Supplementary Note 1 | Further notes on j_0.

As discussed in the main text, j_0 is a materials specific constant and is negatively correlated with E_g. For ideal diodes based on conventional inorganic semiconductors, j_0 can be described by the following equation[2].

$$j_0 = qn_i^2BL \tag{S1}$$

where n_i is the intrinsic carrier concentration, B is the radiative recombination constant, and L is the thickness of the device active layer. Here, n_i is related to E_g according to the following equation.

$$n_i = \sqrt{N_V N_C}e^{-\frac{E_g}{2kT}} \tag{S2}$$

where N_V, N_C are the effective densities of states in the valence and conduction bands, respectively. The quantity B in Eq. (S1) can be described by[3]

$$B = \frac{2}{\tau_m n_M} \tag{S3}$$

where n_M is the majority carrier concentration and τ_m is the minority carrier lifetime. For simplicity, here the donor and acceptor concentrations on the two sides of the junction are assumed to be equal. Minority carrier electron and hole lifetimes, τ_e and τ_h, are assumed to be equal to τ_m. Substituting Eqs. (S2) and (S3) into Eq. (S1) gives:

$$j_0 = \frac{2}{\tau_m n_M} qLN_V N_C e^{-\frac{E_g}{kT}} \tag{S4}$$

It can be seen that j_0 is negatively correlated with E_g. The actual values of j_0 are expected to differ greatly across different materials systems and devices, but it follows the general form below.

$$j_0 = \Lambda e^{-\frac{E_g}{kT}} \tag{S5}$$

where Λ is a quantity affected by materials properties and device design.

Supplementary Note 2 | Derivation of the I_{EL}-V relation.

To accommodate both low- and moderate-voltage ranges where the effect of series resistance (R_s) can not be neglected, the current-voltage (*J*-*V*) characteristics of an LED can be described by

$$j = j_0(e^{\frac{q(V-jR_S)}{nkT}} - 1) \quad \text{(S6)}$$

Inserting Eq. (S6) and into Eq. (2) (of the main text) gives the following:

$$\frac{qI_{EL}}{\mathrm{EQE}} = j_0\left(e^{(\frac{qV}{nkT}-\frac{q^2R_SI_{EL}}{nkT\mathrm{EQE}})} - 1\right) \quad \text{(S7)}$$

Neglecting the minus-one term on the right-hand side, taking natural logarithm on both sides of the equation and rearranging the terms give

$$\ln\left(\frac{qI_{EL}}{\mathrm{EQE}j_0}\right) + \frac{q^2R_s}{nkT\mathrm{EQE}}I_{EL} = \frac{qV}{nkT} \quad \text{(S8)}$$

By inspection, Eq. (S8) takes the form

$$\ln(A + Bx) + Cx = D \quad \text{(S9)}$$

The Lambert W function gives the following solution:

$$x = -\frac{A}{B} + \frac{1}{C}\mathrm{W}\left(\frac{C}{B}e^{\frac{AC}{B}+D}\right) \quad \text{(S10)}$$

Given I_{EL} is the variable to be solved, Eq. (S8) is in the standard form of Eq. (S9). The solution for I_{EL} is

$$I_{EL} = \frac{nkT\mathrm{EQE}}{q^2R_s}\mathrm{W}\left(\frac{qR_sj_0}{nkT}e^{\frac{qV}{nkT}}\right) \quad \text{(S11)}$$

Taking the logarithm to Eq. (S11) and invoking $\log W(z) = \log(z) - W(z)\log(e)$ give the relation:

$$\log(I_{EL}) = \frac{q\log(e)}{nkT}V + \log(\mathrm{EQE}) - \log(e)\,\mathrm{W}\left(\frac{qR_sj_0}{nkT}e^{\frac{qV}{nkT}}\right) + \log(j_0/q) \quad \text{(S12)}$$

Supplementary References

1. E. F. Schubert, *Light-Emitting Diodes*. (Cambridge University Press, 2003).
2. Würfel, P. *Physics of solar cells: from principles to new concepts*. (Wiley-Vch, 2005).
3. Casey, H. C. & Panish, M. B. *Heterostructure Lasers*. (Academic Press, 1978).
